# Risk-based triage strategy by extended HPV genotyping for women with ASC-US cytology

**DOI:** 10.1080/07853890.2025.2451183

**Published:** 2025-01-17

**Authors:** Xuan Rao, Yue-Han Wang, Rui-Zhe Chen, Qian-Qian Wu, Xiao-Fei Zhang, Yun-Feng Fu, Xin-Yu Wang, Xiao Li

**Affiliations:** a Department of Gynecologic Oncology, Women’s Hospital, School of Medicine Zhejiang University, Hangzhou, China; b Zhejiang Provincial Key Laboratory of Precision Diagnosis and Therapy for Major Gynecological Diseases, Hangzhou, China; c Medical Centre for Cervical Diseases, Women’s Hospital, School of Medicine Zhejiang University, Hangzhou, China; d Department of Pathology, Women’s Hospital, School of Medicine Zhejiang University, Hangzhou, China; e Department of Obstetrics and Gynecology, The First Affiliated Hospital Zhejiang University School of Medicine, Hangzhou, China; f Zhejiang Provincial Clinical Research Center for Obstetrics and Gynecology, Hangzhou, China

**Keywords:** Atypical squamous cells of undetermined significance, human papillomavirus, extended genotyping, colposcopy, cervical intraepithelial neoplasia, risk-based triage strategy

## Abstract

**Objective:**

We attempted to evaluate the immediate high-grade squamous intraepithelial lesion-cervical intraepithelial neoplasia grade 2/3 or worse (HSIL-CIN2+/3+, hereafter referred to as CIN2+/3+) risk of specific human papillomavirus (HPV) genotype and form the precise risk-based triage strategy for atypical squamous cells of undetermined significance (ASC-US) women.

**Methods:**

The clinical data of ASC-US women who underwent HPV genotyping testing and colposcopy were retrospectively reviewed. The distribution and CIN2+/3+ risks of specific HPV genotype were assessed by three approaches. The risk-based triage strategy was further established, and its efficacy in detecting CIN2+/3+ was estimated.

**Results:**

Totally, 5553 ASC-US women including 3648 HPV-positive and 1905 HPV-negative were analysed. CIN2+/3+ were 662/319 cases, including 639/306 HPV-positive and 23/13 HPV-negative women. HPV16, HPV52, HPV58 and HPV18 were always among the top 5 ranking genotypes, no matter in HPV-positive women or in HPV-positive CIN2+/3+ cases. HPV16 and HPV33 carried the highest risk, while HPV73 and 26 carried the least risk for CIN2+/3+. Based on the immediate CIN2+/3+ risk of specific HPV genotype, 18 HPVs were divided into three risk-stratified groups. Only women infected with HPVs included in group A were necessary for immediate colposcopy. Compared with conventional strategy, this new risk-based strategy not only had higher specificity (CIN2+: *p* = .00; CIN3+: *p* = .01) and positive predictive value (CIN2+: *p* = .00; CIN3+: *p* = .03) for detecting CIN2+/3+, but also needed fewer colposcopies to identify each CIN2+/3+.

**Conclusions:**

A new triage strategy for ASC-US women was successfully constructed based on CIN2+/3+ risks of 14 high-risk and 4 intermediate-risk HPVs, which could significantly reduce unnecessary colposcopies.

## Introduction

Cervical cancer remains a high burden on global public health with the fourth highest incidence and mortality in women [1]. Effective screening methods such as cytology and high-risk human papillomavirus (hr-HPV) testing have been shown to be successful in reducing cervical cancer mortality by detecting precancers. Nowadays, cervical cancer screening strategy has gradually changed from cytology-based testing to HPV-based testing in China. Atypical squamous cells of undetermined significance (ASC-US) cytology is the most common cervical cytological abnormality, accounting for approximately 44.9–67.5% in China [2–4] and 44.4–67.4% in other countries [5–8]. All hr-HPV-positive women with ASC-US should be referred for colposcopy as a conventional triage strategy, since immediate risks of high-grade squamous intraepithelial lesion-cervical intraepithelial neoplasia grade 2/3 or worse (HSIL-CIN2+/3+, hereafter referred to as CIN2+/3+) were 10.2–35.7% and 4.3–8.4%, respectively [9–15].

However, the CIN2+/3+ risk of ASC-US varied among different HPV genotype and different detective methods [10,11,15–17]. A previous study using hr-HPV mRNA detection [10] found that only HPV16 and 18/45 carried CIN3+ risk >4% among 3052 ASC-US women. But another study using BD Onclarity HPV assay [11] found that HPV16, HPV31, HPV33/58, HPV18, HPV52, HPV51 and HPV35/39/68 carried CIN2+ risks >4% (ranging from 29.5 to 5.4%), while only HPV16, HPV31 and HPV52 carried CIN3+ risks >4% (ranging from 16.1 to 4.1%) among 1953 ASC-US women. These results suggested some non-16/18 hr-HPVs might carry relatively lower immediate CIN2+/3+ risks than others. Thus, it is necessary to type non-16/18 hr-HPVs for ­better precise risk stratification.

Recently, numerous extended HPV genotyping kits have been approved by National Medical Products Administration (NMPA) in China, which could report each hr-HPV (16, 18, 31, 33, 35, 39, 45, 51, 52, 56, 58, 59, 66 and 68) and some intermediate-risk HPV type (ir-HPV (26, 53, 73 and 82)) individually. Thus, it makes comprehensive risk assessment of each HPV type become a possibility. To the best of our knowledge, the study on CIN2+ risks of hr-HPVs and ir-HPVs was rare [18,19], and no study was reported on the CIN3+ risk using extended HPV genotyping kits among ASC-US women up to date. Therefore, we analysed immediate CIN2+/3+ risks of specific hr-HPV and ir-HPV genotype, and tried to develop the precise risk-based triage strategy among ASC-US women according to the principle of ‘equal management for equal risks’ in American Society for Colposcopy and Cervical Pathology (ASCCP) guidelines [20].

## Materials and methods

### Study design

The clinical information was retrospectively reviewed for the patients who received colposcopy in the Women’s Hospital School of Medicine Zhejiang University between 27 October 2014 and 9 December 2021. Women who met all the following criteria were included: ASC-US cytology, HPV negative or positive with genotyping information, colposcopy was performed. Exclusion criteria were as follows: no biopsy or endocervical curettage during colposcopy, or a history of cervical cancer, or a history of total hysterectomy. The present study was approved by the Ethics Committee of the hospital and adhered to the Declaration of Helsinki (IRB-20220309-R). Owing to the retrospective character of the study, informed consent was specifically waived by the ethics committee.

Liquid-based cytology was performed for all women. And all cytology results were interpreted according·to·the·2014 Bethesda System [21]. HPV infections were detected by full-genotyping assays approved by NMPA, which could report 14 hr-HPVs and four ir-HPVs individually. For the convenience of analysis, hr-HPV and/or ir-HPV-positive was further defined as ‘HPV-positive’ in the present study. Colposcopy was performed for all women by a professional gynaecologist in the Women’s Hospital School of Medicine Zhejiang University.

Cervical histology was diagnosed by the pathologists in our hospital according to the clinical guideline of China [22]. HSIL-CIN2, HSIL-CIN3, adenocarcinoma *in situ*, squamous cell carcinoma and adenocarcinoma together were defined as CIN2+, while HSIL-CIN3 or worse was defined as CIN3+.

### Statistical analysis

All statistical analyses were performed using SPSS 25.0 Statistics (IBM Corp., Armonk, NY). Pearson’s *χ*^2^ test was used to compare qualitative data. All *p* values were two-sided and *p* < .05 was considered statistically significant. The distribution of HPV genotype was calculated by three approaches, including minimum estimate (Min.), any type estimate (Any.) and hierarchical attribution estimate (Hier.) according to the previous study [2]. Min. was calculated only in the single HPV infection population [2]. Any. was calculated in all HPV infection populations, and multiple HPV infections were calculated more than once [2]. Hier. was included to attribute multiple HPV infections to one specific HPV genotype with the highest ranking from Any. [2]. Moreover, immediate CIN2+/3+ risks of specific HPV genotype were further calculated, which equalled the number of CIN2+/3+ related to specific HPV/the number of specific HPV × 100% [2]. The efficacy of conventional and risk-based triage strategies to identify CIN2+/3+ were assessed through sensitivity (the number of CIN2+/3+ cases among ASC-US women who should be referred to colposcopy/the number of CIN2+/3+ cases × 100%), specificity (the number of less than CIN2+/3+ cases among ASC-US women who should not be referred to colposcopy/the number of less than CIN2+/3+ cases × 100%), positive predictive value (PPV; the number of CIN2+/3+ cases among ASC-US women who should be referred to colposcopy/the number of ASC-US women who should be referred to colposcopy × 100%) and negative predictive value (NPV; the number of less than CIN2+/3+ cases among ASC-US women who should not be referred to colposcopy/the number of ASC-US women who should not be referred to colposcopy). In addition, the number of colposcopies needed to identify each CIN2+/3+ case was also calculated.

## Results

### Basic characteristics of 5553 women with ASC-US cytology

As shown in Table 1 and Supplemental Figure S1, totally 5553 women were finally enrolled, including 3648 (65.69%) HPV-positive and 1905 (34.31%) HPV-negative women. The median age of 5553 women was 43 years (range: 18–75), including 42 years (range: 18–75) for HPV-positive, and 44 years (range: 21–75) for HPV-negative women. The age-specific distribution of HPV infection exhibited twin-peaks at 30–39 years and at 50–64 years. CIN2+/3+ were 662/319 cases, including 639/306 HPV-positive and 23/13 HPV-negative women. Among HPV-positive women, CIN2+/3+ were 484/239 cases in 2711 single HPV infections and 155/67 in 937 multiple HPV infections. Although single HPV infections were more than multiple HPV infections (74.31 vs. 25.69%), there was no significant difference for CIN2+/3+ risks (*χ*^2^ = 0.83, *p* = .36; *χ*^2^ = 2.51, *p* = .11) (Supplemental Table S1).

**Table 1.** Basic characteristics of 5553 women with ASC-US cytology.

	Total (*n* = 5553)	HPV-positive (*n* = 3648)	HPV-negative (*n* = 1905)
Age, years			
Medium (min–max)	43 (18–75)	42 (18–75)	44 (21–75)
<25	187 (3.37)	158 (4.33)	29 (1.52)
25–29	515 (9.27)	373 (10.22)	142 (7.45)
30–39	1703 (30.67)	1166 (31.96)	537 (28.19)
40–49	1417 (25.52)	847 (23.22)	570 (29.92)
50–64	1561 (28.11)	991 (27.17)	570 (29.92)
≥65	170 (3.06)	113 (3.10)	57 (2.99)
Histology			
<CIN2	4891 (88.08)	3009 (82.48)	1882 (98.79)
CIN2	343 (6.18)	333 (9.13)	10 (0.52)
CIN3	307 (5.53)	296 (8.11)	11 (0.58)
Cancer	12 (0.22)	10 (0.27)	2 (0.10)

ASC-US: atypical squamous cells of undetermined significance; HPV: human papillomavirus; CIN: cervical intraepithelial neoplasia.

### Distribution and the immediate risk of specific HPV genotype

**Figure 1.**
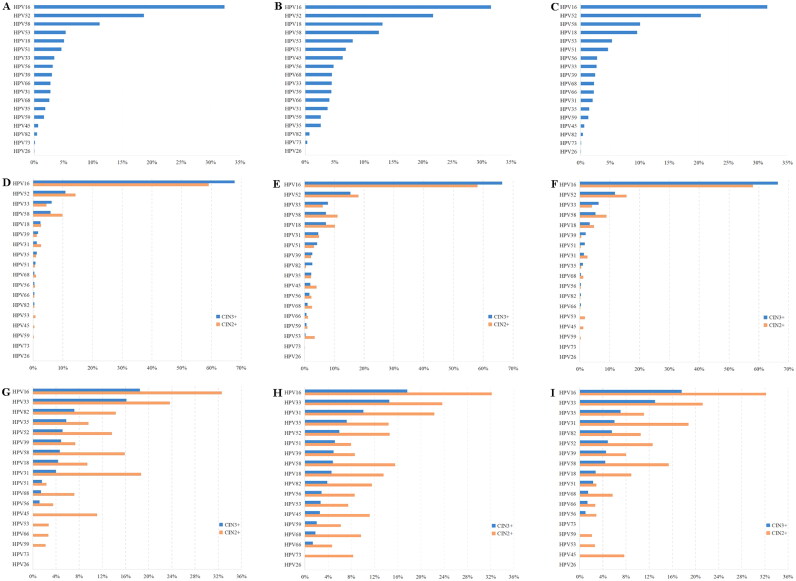
Distribution and the immediate risk of specific HPV genotype. (A) The distribution of specific HPV genotype in HPV-positive ASC-US women estimated by Min. (B) The distribution of specific HPV genotype in HPV-positive ASC-US women estimated by Any. (C) The distribution of specific HPV genotype in HPV-positive ASC-US women estimated by Hier. (D) The distribution of specific HPV genotype in HPV-positive CIN2+/3+ cases estimated by Min. (E) The distribution of specific HPV genotype in HPV-positive CIN2+/3+ cases estimated by Any. (F) The distribution of specific HPV genotype in HPV-positive CIN2+/3+ cases estimated by Hier. (G) The immediate CIN2+/3+ risk of specific HPV genotype in HPV-positive ASC-US women estimated by Min. (H) The immediate CIN2+/3+ risk of specific HPV genotype in HPV-positive ASC-US women estimated by Any. (I) The immediate CIN2+/3+ risk of specific HPV genotype in HPV-positive ASC-US women estimated by Hier. ASC-US: atypical squamous cells of undetermined significance; HPV: human papillomavirus; Min.: minimum estimate; Any.: any type estimate; Hier.: hierarchical attribution estimate; CIN: cervical intraepithelial neoplasia.

Among HPV-positive ASC-US women, HPV16, HPV52, HPV58, HPV18 and HPV53 were always the top 5 ranking genotypes using three approaches (Supplemental Table S2 and Figure 1). The proportion of hr-HPV infection was significantly higher than ir-HPV infection (94.16 vs. 5.84%) using Hier. When the distribution of specific HPV genotype in HPV-positive CIN2+/3+ cases was calculated, HPV16, HPV52, HPV58 and HPV18 were still among the top 5 genotypes but HPV53 was replaced by HPV33 (Supplemental Table S3 and Figure 1). This indicated that the CIN2+/3+ risk of HPV53 was lower than its infective risk, while HPV33 carried higher CIN2+/3+ risk than its infective risk. Moreover, HPV73 and HPV26 were always the last two ranking genotypes, no matter in HPV-positive women or in HPV-positive CIN2+/3+ cases.

As we know, the CIN2+/3+ risk of each HPV genotype could help us to better estimate the carcinogenicity [2]. Herein, we further analysed the immediate CIN2+/3+ risk of specific HPV genotype (Supplemental Table S4 and Figure 1). As shown in Supplemental Table S4, HPV 16 and HPV33 always carried the top 2 highest risk for CIN2+/3+ risks using three approaches. Among 14 hr-HPVs, only HPV56, HPV51, HPV66 and HPV59 carried low CIN2+ risk (<4%), while HPV18, HPV68 and HPV45 also carried low CIN3+ risk (<4%) except aforementioned four hr-HPVs using Hier. Among four ir-HPVs, only HPV82 always carried high CIN2+/3+ risks, while HPV 53, HPV73 and HPV26 always carried low risk using Hier.

### Risk stratification for specific HPV genotype

**Figure 2.**
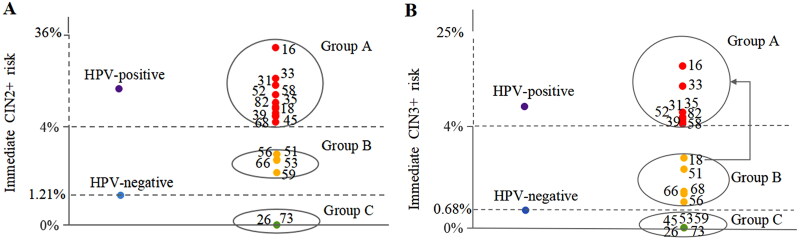
Algorithms of risk stratification for specific HPV genotype grouping in ASC-US women. (A) HPV genotype grouping for CIN2+. HPVs that carried the immediate CIN2+ risk above the threshold of colposcopy referral (≥4.0%) were classified into group A, including HPV16, HPV33, HPV31, HPV58, HPV52, HPV35, HPV82, HPV18, HPV39, HPV45 and HPV68. HPVs that carried the immediate CIN2+ risk no more than that of HPV-negative women were group C, including HPV73 and HPV26. And the others were group B, including HPV56, HPV51, HPV66, HPV53 and HPV59. (B) HPV genotype grouping for CIN3+. According to prior studies, HPV18 was still grouped into A by default due to its critical role in cervical cancer and necessity for colposcopy referral. For remaining HPV genotypes, HPVs that carried the immediate CIN3+ risk above the threshold of colposcopy referral were classified into group A, including HPV16, HPV33, HPV35, HPV31, HPV82, HPV52, HPV39 and HPV58. HPVs that carried the immediate CIN3+ risk no more than that of HPV-negative women were group C, including HPV45, HPV53, HPV59, HPV73 and HPV26. And the others were group B, including HPV51, HPV68, HPV66 and HPV56. ASC-US: atypical squamous cells of undetermined significance; HPV: human papillomavirus; Min.: minimum estimate; CIN: cervical intraepithelial neoplasia.

**Figure 3.**
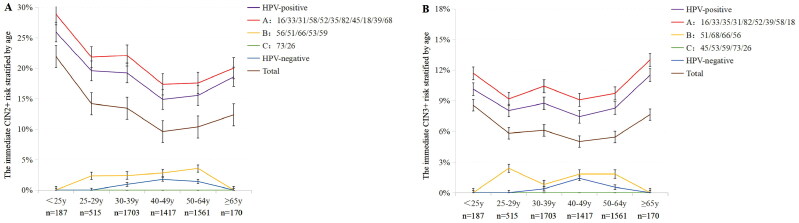
The immediate CIN2+/3+ risk of HPV-positive women, three risk-stratified groups, HPV-negative women and total population stratified by age. (A) The age-specific CIN2+ risk of HPV-positive women, three HPV risk groups, HPV-negative women and total population. (B) The age-specific CIN3+ risk of HPV-positive women, three HPV risk groups, HPV-negative women and total population. CIN: cervical intraepithelial neoplasia; HPV: human papillomavirus.

Referring to the principle of ‘equal management for equal risks’ in the ASCCP guideline, 18 HPVs were further stratified into three groups by the immediate CIN2+/3+ risks using Hier (Table 2 and Figure 2). HPVs that carried the CIN2+/3+ risk above the ASCCP threshold of colposcopy referral (≥4.0%) were classified into group A. HPVs that carried the CIN2+/3+ risk no more than that of HPV-negative women were group C. And the others were group B. Although the CIN3+ risk of HPV18 was <4%, HPV18 was still classified into group A by default due to its critical role in cervical cancer and necessity for colposcopy referral according to previous studies [23,24]. When immediate CIN2+/3+ risks of three groups stratified by age were further evaluated (Supplemental Table S5 and Figure 3), the results remained unchanged. Thus, according to ASCCP guidelines, group A was recommended for colposcopy due to the high immediate risk. Group B was suggested to be followed up for 1 year or further stratified by additional triage test such as p16/Ki-67 dual stain (DS) or DNA methylation detection, since the immediate CIN2+/3+ risks were already higher than 0.55%. Due to the extremely low risk, group C was recommended to be managed as HPV-negative ASC-US women (Table 2).

**Table 2.** The risk-based triage strategy based on HPV genotype for ASC-US cytology^a^.

Group			*N*	Immediate risk, *n*%	Management^b^
CIN2+	A	16/33/31/58/52/35/82/18/39/45/68	3124	625 (20.01)	Colposcopy
	B	56/51/66/53/59	519	14 (2.71)	1-Year follow-up or additional triage test
	C	73/26	5	0 (0)	As HPV-negative
CIN3+	A	16/33/35/31/82/52/39/58/18	3016	300 (9.95)	Colposcopy
	B	51/68/66/56	410	6 (1.46)	1-Year follow-up or additional triage test
	C	45/53/59/73/26	222	0 (0)	As HPV-negative

ASC-US: atypical squamous cells of undetermined significance; HPV: human papillomavirus; CIN: cervical intraepithelial neoplasia.

^a^ According to prior studies, HPV18 was still classified into group A by default due to its critical role in cervical cancer and necessity for colposcopy referral. For remaining HPV genotypes, HPVs that carried the risk above the threshold of colposcopy referral (≥4.0%) were classified into group A, HPVs that carried the risk no more than that of HPV-negative women were group C, and the others were group B.

^b^ According to ASCCP guidelines, HPVs in group A were recommended for immediate colposcopy. Considering that the immediate CIN2+ risk was already higher than 0.55%, HPVs in group B were suggested to be followed up for 1 year or further stratified by additional triage test such as p16/Ki-67 dual stain (DS) or DNA methylation detection. Due to the extremely low risk, group C was recommended to be managed as HPV-negative ASC-US women.

### Efficacy of the conventional and risk-based triage strategy

As the conventional triage strategy, all HPV-positive ASC-US women should be referred for colposcopy. However, only women infected with HPVs included in group A are necessary for immediate colposcopy referrals according to our new risk-based triage strategy. Thus, we found that the risk-based strategy had higher specificity (CIN2+: 38.48 vs. 48.91%, *χ*^2^ = 108.08, *p* = .00; CIN3+: 36.15 vs. 48.11%, *χ*^2^ = 153.55, *p* = .01) and PPV (CIN2+: 17.52 vs. 20.01%, *χ*^2^ = 6.87, *p* = .00; CIN3+: 8.39 vs. 9.95%, *χ*^2^ = 4.85, *p* = .03) than the conventional strategy for detecting CIN2+/3+ (Table 3). This strategy also could reduce the number of colposcopies needed for identifying each CIN2+/3+ case (CIN2+: 5.71 vs. 5.00, *χ*^2^ = 6.87, *p* = .00; CIN3+: 11.92 vs. 10.05, *χ*^2^ = 4.85, *p* = .03).

**Table 3.** Efficacy of conventional strategy and risk-based triage strategy^a^.

	Sensitivity	Specificity	PPV	NPV	*N*/colposcopy
Conventional					
CIN2+	96.53% (94.75–97.73)	38.48% (37.11–39.86)	17.52% (16.30–18.80)	98.79% (98.16–99.22)	5.71
CIN3+	95.93% (92.96–97.72)	36.15% (34.85–37.47)	8.39% (7.52–9.35)	99.32% (98.80–99.62)	11.92
Risk-based					
CIN2+	94.41% (92.31–95.98)	48.91% (47.50–50.32)	20.01% (18.63–21.46)	98.48% (97.88–98.91)	5.00
CIN3+	94.04% (90.70–96.28)	48.11% (46.75–49.47)	9.95% (8.91–11.08)	99.25% (98.81–99.54)	10.05

CIN: cervical intraepithelial neoplasia; PPV: positive predictive value; NPV: negative predictive value.

^a^ The conventional strategy meant all HPV-positive women should be referred for colposcopy, while the risk-based triage strategy meant only women infected with HPVs included in group A are necessary for immediate colposcopy referral.

## Discussion

In the present study, the age-specific distribution of HPV infections exhibited twin peaks at 30–39 years (31.96%) and at 50–64 years (27.17%), which was similar to previous reports [19, 25]. Previous studies reported that HPV16, HPV52 and HPV58 [18,19,26] were the most frequent hr-HPVs, and HPV53 [18,19] was the most frequent ir-HPV among ASC-US women. Similar to these studies, our results showed that HPV16, HPV52, HPV58, HPV18 and HPV53 were the most common genotypes among ASC-US women using any calculating methods. Moreover, the genotypic distribution of HPV is also age-dependent. Ma et al. [27] investigated the age-stratified distribution of specific HPV genotype among women in China. For HPV-positive women, HPV16 was consistently the most common genotype in all age groups (≤25, 26–35, 36–45, 46–55, >55), while HPV59 was more common at ≤25 years, HPV56 was more common at >55 years than other age groups [27]. Similarly, for HPV-positive ASC-US women in the present study, HPV16 was also the most common genotype in all age groups and HPV56 was more common at ≥65 years, while HPV51 was more common at <25 years than other age groups (Supplemental Table S6). Interestingly, HPV53 ranked significantly lower in HPV-positive CIN2+/3+ cases than in HPV-positive women, which suggested that its carcinogenic effect was relatively low. While HPV82 seemed to carry higher carcinogenic risk than many other hr-HPVs. Moreover, HPV73 and HPV26 always ranked last, no matter in HPV-positive women or in HPV-positive CIN2+/3+ cases. Thus, we deduced that HPV82 and HPV53 might have similar importance as hr-HPVs in cervical cancer screening.

Consistent with previous reports [9–15], the present study showed that the immediate CIN2+/3+ risks of hr-HPV-positive ASC-US were 17.52% and 8.39%, respectively. Based on these results, the conventional triage strategy suggested that all hr-HPV-positive ASC-US women should be referred for colposcopy. However, the CIN2+/3+ risks of different HPV genotype in ASC-US women were varied in previous reports [15,18,19], which indicated that some colposcopy referrals might be unnecessary. So, we further analysed and compared the risks of specific HPV genotype reported in previous studies. All data from US and China suggested that HPV16 always carried the highest risk among all hr-HPVs [11,15,18,19,28]. And most studies suggested that high-risk HPV18, HPV31, HPV33, HPV52 and HPV58 also carried high CIN2+ (7.0–30.0%) [11,18,19] or CIN3+ risk (4.1–16.0%) [11, 15], and intermediate-risk HPV82 carried high CIN2+ (14.9–30.8%) [18,19]. But risks of HPV45 and HPV35 were inconsistent in different studies. For example, Jiang et al. [18] found that HPV45 carried high CIN2+ risk (24.2%), while Wright et al. [11] had the opposite result (3.1%). Nevertheless, these data suggested that the different risk of specific HPV genotype might provide the risk stratification in cervical cancer screening. Unfortunately, all previous studies did not use the same threshold to assess the risk of specific HPV genotype. Therefore, we innovatively attempted to construct the risk-based triage strategy for ASC-US women based on ‘equal management for equal risks’ raised by ASCCP in 2019.

In the present study, we adopted the immediate CIN2+/3+ risk of 4.0% as the threshold for colposcopy referral and the risk stratification model was successfully constructed. Similar with previous reports [18,19], our results suggested only 10 hr-HPVs and one ir-HPV carried high CIN2+ risk (>4%), while eight hr-HPVs and one ir-HPV carried high CIN3+ risk (>4%), which met the threshold for colposcopy referral (Table 2). Recently, the newly published guideline of DS testing with CINtec *PLUS* Cytology to triage hr-HPV positive results has suggested that colposcopy is recommended for individuals testing HPV-positive with ASC-US and positive for DS; otherwise, a 1-year return is recommended if negative for DS [29]. In addition, as a promising molecular-based technology, previous studies indicated that DNA methylation detection could be applied in cervical cancer screening [30–33]. It was reported that the GynTect^®^ assay composed of six markers (ASTN1, DLX1, ITGA4, RXFP3, SOX17 and ZNF671) had high sensitivity and specificity to triage hr-HPV positive women (for CIN3+: 66.7% and 84.1%) [30]. A novel marker PCDHGB7 was also reported with the ability to triage hr-HPV positive women, with sensitivity of 82.4% and specificity of 91.1% for CIN2 + [33]. Therefore, for HPVs that carried the immediate CIN2+/3+ risks between the risk of HPV-negative women and 4%, further another triage test such as DS or DNA methylation detection was also recommended in addition to 1-year follow-up. To the best of our knowledge, there was no study on the CIN3+ risk evaluation of ir-HPVs among ASC-US women. Our results suggested HPV82 carried high CIN2+/3+ risks, which implied that HPV82 might deserve further individual detection by FDA-approved HPV kits. Moreover, when compared with the conventional strategy, our risk-based triage strategy reduced the rate of immediate colposcopy referral by 16.45% for CIN2+ and 19.36% for CIN3+. And the sensitivity and NPV remained high as the conventional strategy. Therefore, we thought that the present risk-based stratification could provide more precise management for HPV-positive ASC-US women.

With wider HPV vaccination coverage, the prevalence of vaccine-targeted HPVs would fade away. The extended HPV genotyping could provide the possibility for full assessment of the HPV infection pattern and the epidemiology of cervical lesions. And we believe this risk-based triage strategy based on extended HPV genotyping would be gradually implemented to the clinical protocol for cervical cancer screening in the future. However, the present study still has some limitations. First, as all retrospective studies, there was selective bias. In order to minimize the bias, all ASC-US women who met the criteria were analysed including HPV-positive and HPV-negative women in the present study. However, the risk estimates obtained in this study could only roughly represent the population in this region due to various ethnicities and geographical variation. Therefore, further prospective study in general screening population is needed to confirm our results in the future. Second, the sample size of HPV82, HPV26 and HPV73 infections was small, which made it difficult to assess their pathogenic risk accurately. In addition, there were no data regarding the impact of vaccination on the genotypic distribution of HPV in the present study. Further investigation is needed to understand the influence of vaccination on the distribution of HPV genotypes, the recurrence of the disease and persistent infections in post-vaccination era.

## Conclusions

The present study stratified 14 hr-HPVs and four ir-HPVs into three groups based on CIN2+/3+ risks among ASC-US according to the risk threshold of ASCCP guidelines. A new risk-based stratification strategy was constructed, which had higher specificity and PPV for ASC-US triage and significantly reduced unnecessary colposcopies. However, more detailed investigation is urgently needed worldwide. In the future, we will further verify our results in a large-sample cervical cancer screening cohort and add the evidence of cumulative risks of specific HPV genotype among ASC-US women.

## Supplementary Material

Figure_and_supplementary_figure_legends_clean.doc

## Data Availability

The primary data are available from the corresponding author on reasonable request.
