# Supplementary material for: Risk-based triage strategy by extended HPV genotyping for women with ASC-US cytology
Source: Ann Med. 2025 Jan 17;57(1):2451183. doi: 10.1080/07853890.2025.2451183 (PMC11749152; doi:10.1080/07853890.2025.2451183)
Supplement: Figure_and_supplementary_figure_legends_clean.doc [file IANN_A_2451183_SM9674.doc]

**Figure 1.** Distribution and the immediate risk of specific HPV genotype.

(A) The distribution of specific HPV genotype in HPV-positive ASC-US women estimated by Min. (B) The distribution of specific HPV genotype in HPV-positive ASC-US women estimated by Any. (C) The distribution of specific HPV genotype in HPV-positive ASC-US women estimated by Hier. (D) The distribution of specific HPV genotype in HPV-positive CIN2+/3+ cases estimated by Min. (E) The distribution of specific HPV genotype in HPV-positive CIN2+/3+ cases estimated by Any. (F) The distribution of specific HPV genotype in HPV-positive CIN2+/3+ cases estimated by Hier. (G) The immediate CIN2+/3+ risk of specific HPV genotype in HPV-positive ASC-US women estimated by Min. (H) The immediate CIN2+/3+ risk of specific HPV genotype in HPV-positive ASC-US women estimated by Any. (I) The immediate CIN2+/3+ risk of specific HPV genotype in HPV-positive ASC-US women estimated by Hier.

Abbreviations: ASC-US, atypical squamous cells of undetermined significance; HPV, human papillomavirus; Min., minimum estimate; Any., any type estimate; Hier., hierarchical attribution estimate; CIN, cervical intraepithelial neoplasia.

**Figure 2.** Algorithms of risk stratification for specific HPV genotype grouping in ASC-US women.

(A) HPV genotype grouping for CIN2+. HPVs that carried the immediate CIN2+ risk above the threshold of colposcopy referral (≥4.0%) were classified into group A, including HPV16, HPV33, HPV31, HPV58, HPV52, HPV35, HPV82, HPV18, HPV39, HPV45, and HPV68. HPVs that carried the immediate CIN2+ risk no more than that of HPV-negative women were group C, including HPV73 and HPV26. And the others were group B, including HPV56, HPV51, HPV66, HPV53, and HPV59. (B) HPV genotype grouping for CIN3+. According to prior studies, HPV18 was still grouped into A by default due to its critical role in cervical cancer and necessity for colposcopy referral. For remaining HPV genotypes, HPVs that carried the immediate CIN3+ risk above the threshold of colposcopy referral were classified into group A, including HPV16, HPV33, HPV35, HPV31, HPV82, HPV52, HPV39, and HPV58. HPVs that carried the immediate CIN3+ risk no more than that of HPV-negative women were group C, including HPV45, HPV53, HPV59, HPV73, and HPV26. And the others were group B, including HPV51, HPV68, HPV66, and HPV56.

Abbreviation: ASC-US, atypical squamous cells of undetermined significance; HPV, human papillomavirus; Min., minimum estimate; CIN, cervical intraepithelial neoplasia.

**Figure 3.** The immediate CIN2+/3+ risk of HPV-positive women, 3 risk-stratified groups, HPV-negative women and total population stratified by age.

(A) The age-specific CIN2+ risk of HPV-positive women, 3 HPV risk groups, HPV-negative women and total population. (B) The age-specific CIN3+ risk of HPV-positive women, 3 HPV risk groups, HPV-negative women and total population.

Abbreviations: CIN, cervical intraepithelial neoplasia; HPV, human papillomavirus.

Figure S1. The flow chart of women included in this study.

Abbreviations: ASC-US, atypical squamous cells of undetermined significance; HPV, human papillomavirus; CIN, cervical intraepithelial neoplasia.
